# Correlations of gene polymorphisms of angiotensin-converting enzyme 2 with onset and prognosis of hypertrophic cardiomyopathy

**DOI:** 10.5937/jomb0-54585

**Published:** 2025-03-21

**Authors:** Sujuan Li, Xizhi Lin, Haoting Ye, Biyun Li, Siyu Zeng, Qinghua Mei, Yukai Huang, Yixuan Liu

**Affiliations:** 1 The Affiliated Guangdong Second Provincial General Hospital of Jinan University, Department of Pharmacy, Guangzhou, China; 2 Guangdong University of Technology, Guangzhou, China; 3 The Affiliated Guangdong Second Provincial General Hospital of Jinan University, Department of Rheumatology and Immunology, Guangzhou, China; 4 The Affiliated Guangdong Second Provincial General Hospital of Jinan University, Department of Cardiology, Guangzhou, China

**Keywords:** hypertrophic cardiomyopathy, ACE2, polymorphisms, hipertrofična kardiomiopatija, ACE2, polimorfizmi

## Abstract

**Background:**

To investigate the correlations of the gene polymorphisms of angiotensin-converting enzyme 2 (ACE2) with the onset and prognosis of hypertrophic cardiomyopathy (HCM), so as to provide references for the early prevention and precise treatment of HCM in the future.

**Methods:**

In this case-control study, a total of 100 HCM patients (HCM group) and 100 healthy people receiving physical examination who had matched age, gender and race (Control group) were collected. The single nucleotide polymorphisms (rs102312, rs102883 and rs119247) in the promoter region of ACE2 gene were genotyped by means of conformation-difference gel electrophoresis. Whether the distribution frequency of ACE2 genotypes is in agreement with the law of genetic equilibrium was examined using chi-square test. Meanwhile, the correlations of different polymorphisms and alleles in the promoter region of ACE2 gene with the onset and prognosis of HCM were analyzed.

**Results:**

The Hardy-Weinberg equilibrium analysis showed that all the three polymorphisms of ACE2 gene were in agreement with the law of genetic equilibrium (p>0.05). According to the results of genetic association analysis, the polymorphism rs102312 and its alleles in the promoter region of ACE2 gene were correlated with the occurrence of HCM (p<0.05), while the polymorphisms rs102883 and rs119247 as well as their alleles had no associations with the occurrence of HCM (p>0.05). Furthermore, it was found that the cardiac function and prognosis of HCM patients carrying genotype GG of polymorphism rs102312 were poorer than those of patients carrying genotype TT (p<0.05).

**Conclusions:**

The polymorphism rs102312 in the promoter region of ACE2 gene is associated with the onset of HCM in patients, and HCM patients carrying the genotype GG of polymorphism rs102312 have a poorer clinical prognosis.

## Abstract (sr)

**Uvod:**

Cilj je bio da se istraži korelacija polimorfizama gena angiotenzin konvertujućeg enzima 2 (ACE2) sa početkom i prognozom hipertrofične kardiomiopatije (HCM), kako bi se obezbedile reference za ranu prevenciju i precizno lečenje HCM u budućnosti.

**Metode:**

U ovoj studiji slučaj-kontrola, prikupljeno je ukupno 100 pacijenata sa HCM (HCM grupa) i 100 zdravih ljudi koji su bili podvrgnuti fizičkom pregledu koji su odgovarali uzrastu, polu i rasi (kontrolna grupa). Pojedinačni nukleotidni polimorfizmi (rs102312, rs102883 i rs119247) u promotorskom regionu ACE2 gena su genotipizovani pomoću gel elektroforeze konformacione razlike. Da li je frekvencija distribucije ACE2 genotipova u skladu sa zakonom genetske ravnoteže ispitano je korišćenjem hi-kvadrat testa. U međuvremenu, analizirane su korelacije različitih polimorfizama i alela u promotorskom regionu ACE2 gena sa početkom i prognozom HCM.

**Rezultati:**

Hardi-Veinbergova analiza ravnoteže je pokazala da su sva tri polimorfizma ACE2 gena u saglasnosti sa zakonom genetske ravnoteže (p>0,05). Prema rezultatima analize genetičke asocijacije, polimorfizam rs102312 i njegovi aleli u promotorskom regionu ACE2 gena su u korelaciji sa pojavom HCM (p<0,05), dok polimorfizmi rs102883 i rs119247 kao i njihovi aleli nisu imali asocijacije sa pojava HCM (p>0,05). Nadalje, utvrđeno je da su srčana funkcija i prognoza pacijenata sa HCM sa genotipom GG polimorfizma rs102312 lošiji od onih kod pacijenata sa genotipom TT (p<0,05).

**Zaključak:**

Polimorfizam rs102312 u promotorskom regionu ACE2 gena povezan je sa pojavom HCM kod pacijenata, a bolesnici sa HCM koji nose genotip GG polimorfizma rs102312 imaju lošiju kliničku prognozu.

## Introduction

Hypertrophic cardiomyopathy (HCM) is a ubiquitous inherited cardiac disease, with no symptoms or symptoms such as angina pectoris, chest tightness, dyspnea, decreased exercise tolerance, syncope, sudden death and heart failure as the major clinical manifestations. Although most patients can survive for a long time, HCM is still one of the most common causes of sudden death of adolescents and athletes [1] [2]. As HCM progresses relatively slowly, it is particularly important to prevent the factors that may result in a poor prognosis. Previous studies have demonstrated that HCM is a kind of autosomal dominant disease induced by gene mutation, whose symptoms do not occur frequently before adulthood. Besides, the majority of patients probably have no clinical manifestations, so hypertension and left ventricular hypertrophy will be detected while the patients visit the doctors due to other diseases or various non-specific cardiovascular system symptoms, bringing great challenges to the clinical diagnosis and treatment of HCM patients [3] [4]. Therefore, it is necessary to further elaborate the genetic pathogenesis of HCM.

The renin-angiotensin-aldosterone system (RAAS), a hormonal cascade system in human body, participates in diverse physiological and pathological processes in human body through humoral regulation and mainly plays vital roles in water-electrolyte balance, blood pressure regulation and cardiovascular remodeling [5]. Classical renin-angiotensin system (RAS) consists of angiotensinogen, angiotensin I (AngI), AngII and angiotensin-converting enzyme (ACE). Among them, ACE can hydrolyze inactive AngI to highly active AngII, and the latter can act on the cardiovascular system [6]. Currently, numerous studies have elucidated that the imbalance of the RAS axis facilitates the cardiovascular remodeling and blood pressure elevation, thus promoting such diseases as heart failure, myocardial infarction and hypertension. As a crucial member of the ACE family, ACE2 has a certain tissue-specific distribution in human body and mainly exists in the heart, blood vessels, kidneys, lungs, liver and testes, and a small amount of ACE2 can be found in the brain, gastrointestinal tract and endometrium [7]. It has been revealed through research that ACE2 gene polymorphisms are related to heart failure, myocardial infarction and hypertension to some extent, but their correlations with HCM have not been reported yet [8].

In this study, we selected the single nucleotide polymorphisms (SNPs) rs102312, rs102883, and rs119247 in the promoter region of the ACE2 gene for analysis. These polymorphisms were chosen due to their strategic location in the promoter region, which has the potential to regulate ACE2 gene expression. Changes in gene expression influenced by these SNPs could play a critical role in the onset and clinical prognosis of hypertrophic cardiomyopathy (HCM). This rationale aligns with the growing evidence linking ACE2 polymorphisms to various cardiovascular conditions, emphasizing the importance of understanding their role in HCM.

## Materials and method

### Subjects

A total of 100 HCM patients with an age of (47.45±2.83) years and complete clinical data, who were diagnosed and treated in our hospital from August 2017 to August 2022, were selected as the HCM group. All enrolled HCM patients were diagnosed in accordance with the 2014 European Society of Cardiology Guidelines for hypertrophic cardiomyopathy. Patients with cardiac hypertrophy triggered by other causes, such as exercise-induced physiological cardiac hypertrophy, rheumatic mitral valve disease, amyloidosis, and Fabry disease, were excluded based on clinical symptoms and echocardiograms. The control group consisted of 100 healthy individuals, aged (48.22±1.23) years, undergoing routine physical examinations during the same period. These controls were matched with the HCM group for age, gender, and ethnicity to reduce potential confounding variables. All participants provided informed consent, and the study was approved by the Ethics Committee of our hospital.

### Laboratory examinations

The venous blood (4 mL) was collected from all the subjects in the early morning after fasting for 8 h. Then total cholesterol (TC), triglyceride (TG), high-density lipoprotein cholesterol (HDL-C), low-density lipoprotein cholesterol (LDL-C) and brain natriuretic peptide (BNP) in the serum were measured using a full-automatic biochemical analyzer.

### Two-dimensional Doppler ultrasound

HCM patients were subjected to two-dimensional color Doppler echocardiography using a GE Vivid E9 cardiovascular ultrasound system. The ventricular septal thickness was determined, and HCM was classified based on the site of left ventricular hypertrophy according to the Maron’s classification. All results of echocardiography were processed independently by one attending sonographer with high qualification who was blinded to other clinical data.

### Deoxyribonucleic acid (DNA) extraction

The EDTA-anticoagulated blood (4 mL) was collected from patients in both groups to extract the genomic DNAs according to the instructions of DNA extraction kit (Wuhan Servicebio Technology Co., Ltd., Wuhan, China). Then 2 μL of the extracted DNAs were used to measure the quality in 1.5% agarose gel electrophoresis, and their concentration was detected by virtue of an ultraviolet spectrophotometer at the same time.

### Polymerase chain reaction (PCR) amplification

Primers for polymorphisms rs102312, rs102883 and rs119247 in the promoter region of ACE2 gene were designed separately for amplification. The PCR amplification was performed in a 20 μL system (2.0 μL of DNA templates, 10.0 μL of 2× Mix, 0.4 μL of forward primers, 0.4 μL of reverse primers and 7.2 μL of ddH_2_O) under the following conditions: (95°C for 120 s, 94°C for 30 s, 57°C for 90 s and 72°C for 60 s) × 35 cycles, followed by extension at 72°C for 10 min. Next, the amplification of the gene fragments was examined via the agarose gel electrophoresis. All the primers and product sizes of PCR were listed in Table 1.

**Table 1:** Primer sequences and product sizes of different polymorphisms in the promoter region of ACE2 gene.

Polymorphism	Primer sequence (5’-3’)	Product (bp)
rs102312	Forward: CATCTATATGCTAGCTGATGCTReverse: ATAGTCGATGTC- GATGTCGTAC	253
rs102883	Forward: CTCGTAGCTAAGTCGAACGTCCReverse: ATAGCTAGCT- GACTGTGTGTCA	122
rs119247	Forward: CTAGCTAGTCGTGTGTGTAGTGReverse: ATCGTACGTC- GATCGACTGTGT	220

### Ligase detection reaction

The upstream and downstream probes applied in the reaction were designed and synthesized by BGI. All the upstream probes were prepared into a probe mixture at a concentration of 12.5 pmol/μL after modification by 5’ terminal phosphorylation. Then the ligase detection reaction was conducted using a 3.05 μL system including 0.05 μL of ligases, 1 μL of buffer solution, 1 μL of PCR products and 1 μL of probe mixture under the PCR amplification conditions as follows: 95°C for 120 s, 94°C for 15 s and 50°C for 25 s for a total of 30 cycles. After that, the concentration was measured using the ultraviolet spectrophotometer. Subsequently, BGI was entrusted to accomplish the sequencing of target genes and fragment analysis. All the data were analyzed using GeneMapper. The probe sequences and product sizes of ligase reaction for different ACE2 polymorphisms were shown in Table 2.

**Table 2:** Probe sequences of ligase reaction and product sizes of different TLR3 gene polymorphisms.

Polymorphism	Probe	Probe sequence (5’-3’)	Product (bp)
rs102312	rs102312rs102312-Trs102312-G	P-CGTAGTCAAGTTTTTTTTTTTTTTTTTTT- FAMTTTTTTACGATGCTAGTCGAATTTTTTTT- TATTTTTTTTTTTTTACGATGTGCATTTTTTTTTAAA	183
rs102883	rs102883rs102883-Ars102883-C	P-ATAGTCGTAGTCGTTTTTTTTTTTTTTTTT- FAMTTTTTTTTTTTTTTTTTTTTATCGTAGTCG- TAGTCTTTTTTTTTTTTTTTTTTTTACGTAGTCGATACG	147
rs119247	rs119247rs119247-Ars119247-G	P-AAGCTGATGTCGTAGTTTTTTTTTTTTT- FAMTTTTTTTTTTTTTTTTTCAGCTGATGCTGAT- GCTTTTTTTTTTTTTTTAGCTGATGTCGATGCTGAT	179

### Statistical analysis

Statistic Package for Social Science (SPSS) 22.0 (IBM, Armonk, NY, USA) was adopted to analyze all the data. The enumeration data were presented as frequency and percentage, and the measurement data were expressed by mean ± standard deviation. Chi-square test was used for examination and multiple comparisons of enumeration data, and the measurement data were subjected to *t*-test and analysis of variance. The Hardy-Weinberg equilibrium (HWE) analysis was performed using the chi-square test to evaluate whether the observed genotype frequencies for each polymorphism were consistent with expected frequencies under genetic equilibrium. Adherence to HWE is an important criterion for ensuring that the studied population is representative and free from selection bias or genotyping errors. In this study, the results confirmed that all three polymorphisms (rs102312, rs102883, and rs119247) in the ACE2 gene adhered to HWE, with p-values greater than 0.05, validating the reliability of the genotype distributions in both the HCM and control groups. *p*<0.05 suggested that the difference was statistically significant.

## Results

### Comparisons of basic clinical data between the two groups

As shown in Table 3, there were no statistical differences in the age, sex ratio, smoking, drinking, BMI and TG between the two groups (*p*>0.05). HCM group exhibited significantly higher heart rate, BNP, fasting blood glucose, blood pressure (systolic blood pressure and diastolic blood pressure) and blood lipid level, larger left atrial diameter (LAD) and left ventricular end-diastolic diameter (LVEDD) (*p*<0.05), and significantly lower ejection fraction (EF%) and fractional shortening (FS%) than Control group (*p*<0.05).

**Table 3:** Comparisons of general clinical data and biochemical indexes between HCM group and Control group (mean ± standard deviation). LAD: left atrial diameter, LVEDD: left ventricular end-diastolic diameter, EF: left ventricular ejection fraction, FS: fractional shortening, TG: triglyceride, TC: total cholesterol, HDL-C: high-density lipoprotein cholesterol, LDL-C: low-density lipoprotein cholesterol, BNP: brain natriuretic peptide

Parameter	HCM group (n=100)	Control group (n=100)	*p *
Age (years old)	47.45±2.83	48.22±1.23	0.557
Gender (male/female)	76/ 24	70/ 30	0.789
Smoking (%)	34%	36%	0.621
Drinking (%)	25%	28%	0.349
Heart rate	77±9	68±6	0.015*
BMI	21.84±1.02	21.82±1.32	0.834
BNP	120.23±11.93	78.23±9.23	0.000*
Fasting blood glucose	6.98±2.39	5.43±1.23	0.000*
Systolic pressure (mmHg)	142.93±12.03	123.19±8.34	0.000*
Diastolic pressure (mmHg)	87.23±7.23	74.23±6.02	0.000*
TG (mmol/L)	1.34±0.06	1.24±0.13	0.071
TC (mmol/L)	4.61±0.66	5.32±0.53	0.032*
HDL-C (mmol/L)	1.01±0.41	1.03±0.22	0.724
LDL-C (mmol/L)	2.31±0.672	3.45±1.03	0.001*
LAD (mm)	42.12±2.34	34.92±4.21	0.000*
LVEDD (mm)	46.28±5.23	56.23±4.34	0.000*
EF%	45.23±12.28	65.23±8.23	0.000*
FS%	16.37±5.23	33.92±2.04	0.000*

### Analysis results of ACE2 gene polymorphisms rs102312, rs102883 and rs119247

**Figure 1:**
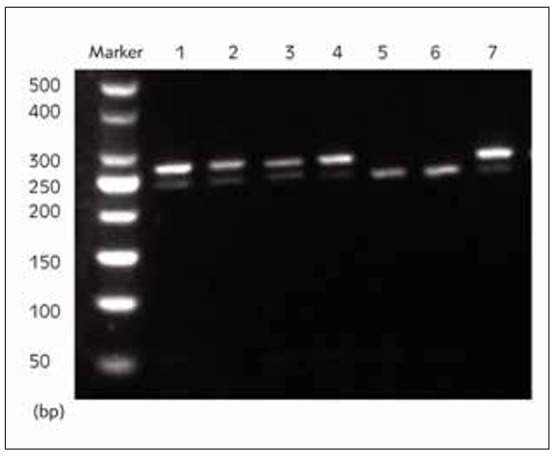
Cleavage sites of ACE2 gene polymorphisms rs102312, rs102883 and rs119247. 1: Genotype TT of polymorphism rs102312, 2: Genotype GG of polymorphism rs102312, 3: Genotype AA of polymorphism rs102883, 4: Genotype CC of polymorphism rs102883, 5: Genotype AA of polymorphism rs119247, 6: Genotype GG of polymorphism rs119247, 7: Amplification products.

After cleavage of ACE2 gene polymorphisms rs102312, rs102883 and rs119247 by restriction enzyme BstU I in both Control group and HCM group, 2 alleles (T and G) and 3 genotypes (TT, TG and GG) of polymorphism rs102312, 2 alleles (A and C) and 3 genotypes (AA, AC and CC) of rs102883, and 2 alleles (A and G) and 3 genotypes (AA, AG and GG) of rs119247 were obtained (Figure 1).

### Hardy-Weinberg equilibrium test results

The results of linkage disequilibrium test of different ACE2 gene polymorphisms were examined through Hardy-Weinberg equation. *r^2^* <0.33 was detected among the polymorphisms in each group, so it was considered that these polymorphisms were in agreement with the equilibrium test (Table 4).

**Table 4:** Results of linkage disequilibrium test of ACE2 gene polymorphisms.

Polymorphism	* r^2^*		
	rs102312	rs102883	rs119247
rs102312	-	0.012	0.293
rs102883	0.012	-	0.138
rs119247	0.293	0.138	-

### Correlation of ACE2 gene polymorphisms with HCM onset

Based on the genotype frequencies of the gene polymorphisms in the two groups (Table 5), the AEC2 gene polymorphism rs102312 was significantly associated with the occurrence of HCM (*p*<0.05). Specifically, the proportion of patients carrying genotype GG of polymorphism rs102312 in HCM group was significantly larger than that in Control group. However, no significant correlations of the polymorphisms rs102883 and rs119247 with the incidence of HCM were observed (*p*>0.05).

**Table 5:** Distribution of different genotypes of ACE2 gene polymorphisms in HCM patients.

Group	rs102312			rs102883			rs119247		
	TT	TG	GG	AA	AC	CC	AA	AG	GG
HCM (n=100)	7%	29%	64%	33%	32%	35%	18%	40%	42%
Control (n=100)	33%	34%	33%	32%	38%	34%	20%	38%	42%
*χ^2^*	1.983			0.238			0.451		
* p *	0.001*			0.892			0.181		

### Correlations of alleles of ACE2 gene with HCM onset

The distribution of different alleles of gene polymorphisms in HCM group and Control group were shown in Table 6. The alleles of the ACE2 gene polymorphism rs102312 had a significant relation to the onset of HCM (*p*<0.05), but the alleles of the polymorphisms rs102883 and rs119247 were not significantly associated with the occurrence of HCM (*p*>0.05).

**Table 6:** Distribution of alleles of ACE2 gene polymorphisms in HCM patients.

Group	rs102312		rs102883		rs119247	
	C	G	A	G	A	T
HCM (n=100)	22.5%	77.5%	49%	61%	38%	62%
Control (n=100)	50%	50%	51%	49%	39%	61%
* χ^2^*	1.873		0.827		0.523	
* p *	0.002*		0.671		0.821	

### Correlations of ACE2 gene polymorphism rs102312 with clinical features and prognosis of HCM patients

Furthermore, the associations of the ACE2 gene polymorphism rs102312 with the clinical features and prognosis of HCM patients were analyzed. The results manifested that among HCM patients carrying genotype GG of ACE2 gene polymorphism rs102312, the ratios of NYHA class ≥3, course of chronic heart failure >1 year and hypertension were higher, and they also had a higher level of serum BNP (*p*<0.05) (Table 7). According to the results of echocardiography (Table 8), the LAD and LVEDD were significantly larger (*p*<0.05), whereas the EF% and FS% were significantly lower (*p*<0.05) in HCM patients with genotype GG of ACE2 gene polymorphism rs102312. It was indicated in the correlation analysis of clinical prognosis that HCM patients with genotype GG of ACE2 gene polymorphism rs102312 exhibited significantly increased incidence rates of major adverse cardiovascular and cerebrovascular event (MACCE), sudden death, stroke and cardiac death compared with those carrying genotype TT (*p*<0.05) (Table 9).

**Table 7:** Correlations of ACE2 gene polymorphism rs102312 with clinical features of HCM patients.

Parameter	NYHA class ≥3 (%)	Course of chronic heart<br>failure >1 year (%)	Hypertension<br>(%)	Coronary heart<br>disease (%)	BNP
TT (n=7)	11.1%	11.1%	28.6%	42.9%	100.23±2.93
TG (n=29)	17.2%	20.7%	37.9%	44.8%	109.45±6.93
GG (n=61)	49.2%	32.8%	65.6%	40.9%	121.34±9.47
* χ^2^*	0.345	0.891	0.231	0.712	1.672
* p *	0.000*	0.000*	0.000*	0.291	0.001*

**Table 8:** Correlation of ACE2 gene polymorphism rs102312 with clinical cardiac function of HCM patients.

Parameter	LAD (mm)	LVEDD (mm)	EF%	FS%
TT (n=7)	36.22±3.23	53.22±4.23	58%	38%
TG (n=29)	41.83±5.99	55.89±3.98	55%	31%
GG (n=61)	46.92±4.37	57.93±1.04	41%	27%
* χ^2^*	2.819	2.398	0.841	2.764
* p *	0.000*	0.000*	0.002*	0.001*

**Table 9:** Correlation of ACE2 gene polymorphism rs102312 with clinical prognosis of HCM patients. *MACCE: major adverse cardiovascular and cerebrovascular event

Parameter	MACCE<br>(%)	Readmission<br>(%)	Decompensated<br>heart failure (%)	Sudden<br>death (%)	Stroke<br>(%)	Cardiac death<br>(%)
TT (n=7)	28.6%	57.1%	49%	0%	14.3%	0%
TG (n=29)	34.5%	51.7%	51%	3.5%	20.7%	3.5%
GG (n=61)	57.4%	54.1%	57.4%	6.6%	32.79%	3.3%
* χ^2^*	1.293	1.893	2.192	0.721	0.883	1.092
* p *	0.001*	0.293	0.109	0.002*	0.028*	0.016

## Discussion

The gene mutation of sarcomeric contractile proteins is the main pathogenic factor of HCM, an autosomal dominant disease, and the morbidity rate of HCM in human is about 1/500, so it is a global disease [9]. HCM is defined as excessive cardiac hypertrophy and a small cavity of the left ventricle that are not caused by other reasons, and some HCM patients may be complicated with cardiac hypertrophy of the right ventricle. HCM can be divided into obstructive type (70%) and non-obstructive type (30%) [10]. According to epidemiologic statistics, HCM is not detected in most patients due to no clinical symptoms, and such patients may have a life-span similar to normal people. However, the symptomatic patients will manifest palpitation, chest pain, exertional dyspnea, syncope, sudden death, etc. Some patients have apparent symptoms, poor cardiac function and quality of life and a high rate of sudden cardiac death [11]. SNP refers to the polymorphism of DNA sequences induced by a single DNA mutation, which is related to multiple complex diseases and phenotypic differences in human. Besides, SNP is one of the most common heritable mutations in human [12]. Generally, it is a dimorphic or biallelic genetic variation composed of four types of bases. Only when the mutation frequency of single nucleotides exceeds 1% can the variation be named SNP. As a kind of genetic marker, SNP is highly stable, and it not only serves as the most reliable basis for finding the cause and diagnosis of disease but also lays a foundation for screening therapeutic drugs [13].

ACE2, a new member of the RAAS discovered in 2000, is the only homologue of human ACE discovered so far, which highly resembles ACE in composition. ACE2 gene is located in human chromosome Xp22, with a length of about 40 kb. There are 3325 bases in the cDNA of ACE2, the full length of mRNA is 3396 bp, and 17 out of 18 exons are highly similar to those of ACE, implying that ACE2 and ACE originate in the same ancestral gene [14]. ACE2 is composed of a signal peptide, a catalytic domain of matrix metalloproteinase and a transmembrane domain. There are 805 amino acids in the whole protein sequences of ACE2. ACE2 is primarily expressed in the heart, kidneys and testes, and it is also distributed in gastrointestinal tract, nervous tissues and lungs [15]. ACE2 is able to convert AngII into Ang-(1-7) that can bind to Mas receptor, a G protein-coupled receptor encoded by proto-oncogene Mas, thus antagonizing the adverse effect of AngII on the cardiovascular system and protecting the cardiovascular system by dilating the blood vessels, decreasing the blood pressure, resisting inflammation, hyperplasia and oxidative stress and facilitating natriuresis [16]. Genetic analysis on rat models indicated that ACE2 gene is located on hypertension-related quantitative trait loci of X chromosome. In transgenic animal models of hypertension, the overexpression of ACE2 can block the occurrence of hypertension, while the knockout of ACE2 gene will increase the susceptibility to AngII-induced hypertension [17]. It has been proven that ACE2 gene plays pivotal roles in RAAS and blood pressure regulation. A survey of Cauca sians with diabetes mellitus demonstrated that the allele C of polymorphism rs2074192, allele G of polymorphism rs4240157 and allele T of polymorphism rs4646188 of ACE2 gene are correlated with hypertension in males, and the allele G of polymorphism rs4240157 has an association with hypertension in females [18]. A study on Korean population revealed that the mutations in ACE2 gene polymorphisms rs1514283 and rs1514282 exhibit significant correlations with the diastolic blood pressure, but they are not significantly associated with the prevalence rate of hypertension. In this study, it was found that the polymorphism rs102312 and its alleles in the promoter region of ACE2 gene had relations to the occurrence of HCM, while the polymorphisms rs102883 and rs119247 as well as their alleles were not associated with the occurrence of HCM. Furthermore, it was discovered that the cardiac function and prognosis of HCM patients carrying genotype GG of polymorphism rs102312 were poorer than those of patients carrying genotype TT. Our findings highlight the potential clinical implications of the rs102312 polymorphism in the ACE2 gene for managing hypertrophic cardiomyopathy (HCM). Identifying this genetic variant could enable early risk stratification, allowing clinicians to identify high-risk individuals before the onset of severe symptoms. Moreover, the association between the GG genotype of rs102312 and poorer clinical outcomes suggests that this polymorphism could serve as a prognostic biomarker to guide personalized treatment strategies. Incorporating genetic screening for ACE2 polymorphisms into routine clinical practice could enhance precision medicine approaches in HCM management.

This study has certain limitations that should be acknowledged. First, the relatively small sample size may reduce the statistical power of our findings and limit the ability to detect associations for less common genotypes. Second, the study focused on a single population, which may restrict the generalizability of our results to other ethnic or geographic groups. These factors highlight the need for future research involving larger and more diverse cohorts to confirm and extend the current findings. Such studies would provide a broader perspective on the role of ACE2 polymorphisms in hypertrophic cardiomyopathy and enhance the translational potential of our findings.

In conclusion, it was confirmed for the first time in this study that the polymorphism rs102312 and its alleles in the promoter region of ACE2 gene are correlated with the incidence and prognosis of HCM to some extent.

## Dodatak

### Funding

This work was supported by the Guangzhou Science and Technology Plan Project - Basic and Applied Basic Research Project - General Project (PhD Young scientists) (Project number: 202201011319) »Title: The role and mechanism of mesenchymal stem cell exosome-mediated activation of Sonic hedgehog pathway in the abnormal accumulation of DNA double-strand breaks in myocardial infarction«; Guangzhou Science and Technology Plan Project - Municipal University (Institute) joint funding project - Basic and applied basic research project (Project number: 202201020309) »Title: overexpression of miR - 125 - b of bone marrow mesenchymal stem cells between exosome effect on the treatment of myocardial infarction and related mechanism research«; Doctor/Doctor workstation project of the Second People’s Hospital of Guangdong Province (Project number: 2021BSGZ021) »Study on the therapeutic effect and related mechanism of EXOSOME of bone marrow mesenchymal stem cells overexpressing MIR-125B on myocardial infarction«.

### Conflict of interest statement

All the authors declare that they have no conflict of interest in this work.
